# Adherence with Preventive Medication in Childhood Asthma

**DOI:** 10.1155/2011/973849

**Published:** 2011-04-06

**Authors:** Scott Burgess, Peter Sly, Sunalene Devadason

**Affiliations:** ^1^Department of Respiratory Medicine, Mater Children's Hospital, QLD 4101, Australia; ^2^Queensland Children's Medical Research Institute, QLD 4029, Australia; ^3^University of Western Australia, WA 6009, Australia

## Abstract

Suboptimal adherence with preventive medication is common and often unrecognised as a cause of poor asthma control. A number of risk factors for nonadherence have emerged from well-conducted studies. Unfortunately, patient report a physician's estimation of adherence and knowledge of these risk factors may not assist in determining whether non-adherence is a significant factor. Electronic monitoring devices are likely to be more frequently used to remind patients to take medication, as a strategy to motivate patients to maintain adherence, and a tool to evaluate adherence in subjects with poor disease control. The aim of this paper is to review non-adherence with preventive medication in childhood asthma, its impact on asthma control, methods of evaluating non-adherence, risk factors for suboptimal adherence, and strategies to enhance adherence.

## 1. Introduction

The aim of this paper is to review non-adherence with preventive medication in childhood asthma and its impact on asthma control. Methods of evaluating non-adherence and risk factors for sub-optimal adherence will be reviewed. Finally, the latest evidence for strategies to enhance adherence will be summarised. 

## 2. Compliance, Adherence, and Concordance

“Compliance” describes the degree to which a patient takes a medication as it has been prescribed [1]. Compliance has largely been replaced by the term “adherence,” which has fewer negative connotations [1]. It has been argued that both terms reflect a paternalistic model of care rather than a partnership. However, the alternative “concordance”, which has been coined to reflect a therapeutic decision that incorporates the common goals of the physician and patient [1], has not been widely accepted. 

## 3. Management of Asthma and Preventive Medication

Preventive medication is the corner stone of treatment for children with frequent intermittent or persistent asthma [2]. Preventive medication is taken on regular basis and has been shown to decrease inflammation within the lung and to improve disease outcomes [3]. 

## 4. Importance of Adherence with Preventive Medication

Non-adherence takes many forms, and the extent to which a patient is adherent with different asthma-related tasks may vary [4]. Patients may fail to attend appointments, fill prescriptions, miss doses of medication, or fail to use their inhalation device correctly. The incorrect use of an asthma device may be accidental (reflecting competence) or deliberate (contrivance) [5].

The impact of non-adherence depends upon the severity of the condition and the effectiveness of the treatment. Non-adherence is not necessarily inappropriate, particularly if the treatment is ineffective or harmful, so-called “intelligent non-adherence” [6]. The consequence of non-adherence with medication may also be dependent upon the pattern of non-adherence and pharmacological characteristics of the medication [7]. Sub-optimal adherence has been shown to result in poor disease control [8–10], an increased risk of hospital admission [11, 12], and asthma-related mortality [13–15]. If non-adherence is undetected, the physician may unnecessarily increase the dose of medication or add an additional treatment, increasing the cost and complexity of the regimen [16]. It has been estimated that non-adherence costs the US healthcare system $300 billion each year [17]. Undetected non-adherence is also important in research trials, skewing results towards the null hypothesis [16]. 

## 5. Measuring and Estimating Adherence with Preventive Medication

Adherence may be measured by collecting data from the child or parent (direct questioning, questionnaires, and diaries), evaluating the amount of medication that has been used (counting returned pills, weighing canisters), reviewing pharmacy records, electronic monitoring devices (incorporated into the pill bottle or pressurised metered dose inhaler), or drug assays (serum or urine drugs levels) [18].

Electronic monitoring devices (EMDs) have been proposed as the Gold standard for measuring adherence [19]. EMDs record a time stamp which can be downloaded and compared with the prescribed regimen. Using these devices, it was discovered that some participants in a clinical trial were discharging large amounts of medication just prior to reviews, a process referred to as “dumping” [20]. EMDs do have limitations, most importantly they are expensive, some devices have been prone to electrical malfunction [18], and monitoring may itself alter the patients' behavior [18].

Compared with EMDs, parental report [21], questionnaires [22], diaries [8, 12, 23, 24], canister weights [21, 22, 25–27], and pill counts [28] have all been found to overestimate adherence. Even when parents were asked about their child's adherence using a nonjudgemental manner, prefaced by a normalising statement, the level of reported adherence did not accurately reflect data from an EMD [29]. Patients typically want their actions to be seen to be appropriate and responsible, a behaviour referred to as “social desirability” [30]. This strong social convention may result in seemingly honest people providing exaggerated reports of medication usage.

Healthcare professionals have been shown to be poor judges of adherence, identifying non-adherence no better than would be predicted by chance alone [29, 31–33]. The inability to predict adherence has been shown to be independent of seniority [34], professional stream (doctors versus nurses) [31], or how long the physician has known the patient [33]. 

## 6. Adherence Rates in Clinical Studies

Studies that have used EMDs to evaluate adherence with preventive asthma medication have reported average adherence rates of 50% to 77% [8, 12, 21, 24, 29, 35]. Even though these figures are low, they are likely to be higher than adherence within the general population as the most nonadherent patients do not normally enroll in trials [36] and simply being in a trial has been associated with increased adherence [37]. 

## 7. Models of Adherence and Behaviour

Models of adherence provide a framework for conceptualising adherence and have been the basis for recommended strategies to manage non-adherence in many review articles. The most commonly cited is the Health Beliefs Model.

According to the Health Beliefs Model, adherence is determined after a patient balances the costs of treatment (e.g., financial costs, and real and feared side effects) and threats posed by the health condition against the perceived benefits of treatment [4]. 

The Ecological Model takes into account contextual factors relating to the family (conflict, poverty, and education), social supports, stressors, and cultural factors. It reminds us that, for some families health may not be the first priority [4]. Other factors may be more pressing, including homelessness, substance abuse, and emotional disturbance [38]. The Ecological Model also takes into account individual factors, such as their beliefs, skills, motivation for change, and self-efficacy [4]. Individual factors also include developmental factors, for example a child's understanding of causality [39].

Evidence suggests that people do not necessarily mentally balance the pros and cons of a given decision. Rather, people typically make decisions instinctively, unconsciously, and are strongly influenced by their emotions and prejudices [40]. 

## 8. Risk Factors and Improving Adherence

### 8.1. Sex and Age

Studies examining adherence in the paediatric population [41], including studies using EMDs, [21, 42] have found no relationship between adherence and gender. The available data indicates that adherence generally falls with increasing age [21, 42–44]. The age at which parents allow children to assume responsibility for taking their own medication may reflect the parents' ability to supervise the child, not only the child's maturity, and this may result in unfounded confidence in their ability [45]. Parents and children may not be aware of the limits of their responsibilities. When the parent and their child believe the other is primarily responsible for remembering to take medication adherence is lower [46]. 

### 8.2. Disease Severity

Patients with frequent symptoms are not necessarily prompted to take their preventive medication. Observational studies have revealed that adherence is lower in those subjects with increased symptoms [35, 42, 44, 47]. Non-adherence should always be suspected in those with poorly controlled asthma. 

### 8.3. Parent-Child Relationship, Parental Psychopathology, and Children's Behaviour

Young children are dependent upon their parents, and, thus, their domestic environment is critical in their disease management [48]. Adolescents report that reminders from parents to take medication are helpful [49, 50], even if they find them annoying [50]. Higher levels of adherence are reported by adolescents who described their parents as supportive and lower when their parents are described as overly anxious or controlling [51].

It is likely that a bidirectional relationship exists between parents' psychological function and their child's asthma control [52]. Children whose parents had poorer mental health scores were reported to experience more asthma symptoms, [53] exacerbations [53], higher rates of health care use [53], and reduced adherence [54]. A meta-analysis of 26 studies found that children with asthma, when compared with controls, have more behavioural difficulties, particularly in terms of internalising behaviours (e.g., anxiety, low mood, withdrawal, and somatic complaints) and to a lesser extent, externalising behaviours (aggressive behaviour or defiance) [55].

Asthmatic children with behavioural difficulties have been found to be less adherent with asthma medication [56]. 

### 8.4. Social Adversity, Ethnicity, and Family Function

Three relatively small, well-designed studies [21, 42, 56] found no relationship between adherence and socioeconomic status. Adherence in these studies was based on either serum drug levels or EMDs. A number of studies in Western countries have found adherence to be lower in non-Caucasian participants [21, 42, 43, 57, 58]. However, minority status is a nonspecific risk factor and could reflect a number of potential barriers to adherence. A study involving African American parents from a low-income urban area reported that the greatest barrier to optimal treatment was parental health beliefs rather than access to healthcare or financial constraints [59]. Family dysfunction has been associated with reduced adherence [12, 21, 56]. Families without strong routines are less likely to fill prescriptions for asthma medication [60]. Non-adherence is greater in families with high levels of conflict and behavioural problems in their children [56]. 

### 8.5. Education, Knowledge and Communication

In order to be adherent patients require basic information about their medication, including how and when they are to be taken. However, numerous studies have failed to correlate asthma knowledge with the level of adherence [21, 42, 60–63], and simply providing information does not necessarily improve adherence [36]. Patients report higher levels of adherence with medication prescribed by physicians who communicate well [57, 58] and whose interactions were described as collaborative rather than authoritarian or generic [51]. In one study, there was 38% disagreement between parents of asthmatic children and their doctor about whether preventive medication had been prescribed during a consultation. Interestingly, discordance was greatest for those parents who had concerns about or negative perceptions of steroid medication [64]. In a landmark study, asthma outcomes were improved when paediatricians were taught to provide simple messages combined with basic communication and counseling strategies [65]. Thus, it is important not merely to educate parents, but to discover and address their specific concerns and confirm that you share common goals and tailor the management plan to the client. 

### 8.6. The Delivery Device

There are limited studies examining the influence of the delivery device on adherence. A breath-activated nebulizer (Halolite) was not found to improve adherence compared with a standard metered dose inhaler and spacer [66]. Adherence was not different when two different dry powder devices were compared [67]. A randomised controlled trial which used an EMD to monitor adherence failed to demonstrate improved adherence by young children using a novel spacer (Funhaler) that included an incentive toy (whistle and spinning disk) [68], contrary to a pilot study that measured adherence using parental report [69]. It is intuitive to offer patients the opportunity to use the device they would prefer, but this does not guarantee increased adherence. 

### 8.7. Frequency of Dosing

There is low-level evidence to suggest that adherence decreases as the dosing frequency of prescribed medication increases [24, 70, 71]. Thus, where possible, the regime should include as few doses as possible. 

### 8.8. Type of Medication

Adherence appears to be higher with a once daily oral nonsteroidal asthma medication compared with an inhaled corticosteroid taken twice daily [72, 73]. The combination of an ICS and long-acting beta-agonist also appears to be associated with superior adherence compared with ICS alone or both medications in separate inhalers [74–76]. However, data on combined medications are not based on studies using EMDs. 

### 8.9. Reminders

The most commonly reported reason patients give for failing to take medication is simply “forgetting” [49, 50, 77]. A randomised controlled study involving adult asthmatics found a significant improvement in adherence in those subjects whose medication was delivered via an EMD which flashed and beeped to remind them of the need to take their medication [78]. 

### 8.10. Measuring Adherence and Providing Feedback

Several studies have found increased adherence when data collected by an EMD were made available to study participants [27, 79–81]. Our group has recently completed a randomised controlled trial involving 26 children with poorly controlled asthma over a 4 month period. Average adherence was 21% higher in those who received feedback on their adherence at each study visit [82]. Larger studies are required to demonstrate the clinical efficacy of this strategy. 

## 9. Conclusion

Suboptimal adherence with preventive medication is common and is associated with significant morbidity and healthcare costs. Non-adherence should be considered in all children with poorly controlled asthma. Adolescents, children from chaotic families, and children from socially disadvantaged groups are at increased risk of non-adherence. Unfortunately, patient report and your own impressions and knowledge of these risk factors may not help you determine whether non-adherence is the reason for a child's poor asthma control. When prescribing medication, it may be important to consider the complexity of the regimen in addition to the efficacy of the intervention. Treatment plans should be developed collaboratively, and it is important to explore the patient's concerns and prejudices. In the future, EMDs are likely to play a greater role as an aide to remind patients to take medication, a strategy to motivate patients to maintain adherence, and a tool to evaluate adherence in subjects with poor disease control. 
